# Interactions of Arachidonic Acid with AAC1 and UCP1

**DOI:** 10.3390/ijms262110504

**Published:** 2025-10-29

**Authors:** Jonathan H. Borowsky, Michael Grabe

**Affiliations:** Cardiovascular Research Institute, Department of Pharmaceutical Chemistry, University of California, San Francisco, San Francisco, CA 94158, USA; jonathan.borowsky@ucsf.edu

**Keywords:** MD simulation, electrostatics, proton uncoupling, AAC1, UCP1

## Abstract

The inner mitochondrial membrane proteins ATP/ADP carrier protein 1 (AAC1) and Uncoupling protein 1 (UCP1) belong to the SLC25 mitochondrial carrier family. AAC1 is responsible for ATP/ADP exchange, while UCP1-dependent proton transport, which also requires small molecules known as activators, is the basis of brown fat thermogenesis. Arachidonic acid (AA) is an endogenous activator capable of inducing proton transport in both proteins. As such, both AAC1- and UCP1-dependent proton transport are potential targets of weight loss drugs. While AAC1 structures have long been available, only recently have structures of UCP1 been determined. Unfortunately, no AA-bound structure of either protein is available. To explore their interactions with AA, we performed molecular dynamics (MD) simulations of both proteins. Six parallel simulations of each protein were run with an average length of just over 6 μs, for a total of 75 μs of aggregate simulation across both proteins. AA bound deeply between transmembrane helix (TM) helices or in the central cavity of AAC1 in 14 events and between TM helices of UCP1 in 6 events. All AA involved in these deep binding events came from the intermembrane space-facing (C) leaflet. In AAC1, AA most often bound between TM1/TM2 and TM5/TM6. In four cases the fatty acid bound at the bottom of the central cavity rather than in an interhelical groove. In UCP1, all but one deeply bound AA sat between TM5 and TM6. No AA fully entered the cavity as observed in AAC1. In addition to entering the proteins, AAs were enriched around them in the surrounding membrane adjacent to the TM helices. While both protein structures exhibit hydrophobic stretches separating the intermembrane space (IMS) from the matrix, water wires formed through both AAC1 and UCP1, connecting the bulk water in both regions. Grotthuss shuttling along water wires has been proposed as a possible mechanism of AAC1/UCP1-dependent proton transport, but water wires are not present in experimental structures and have not previously been reported in MD simulations. Calculations of electric potentials along these water wires find a large 0.75–1 V electrostatic barrier along water wires through AAC1 and a substantially smaller such barrier of ~0.5 V through UCP1.

## 1. Introduction

ATP/ADP carrier protein 1 (AAC1) and Uncoupling Protein 1 (UCP1) are transmembrane proteins in the inner mitochondrial membrane that transport protons across the membrane only in the presence of certain small molecules (termed activators) [1,2,3,4,5,6,7], which are endogenous fatty acids (FA), such as arachidonic acid, or exogenous chemical uncouplers such as 2,4-dinitrophenol (DNP). This transport bypasses ATP synthase, converting the chemical potential of the protons directly into heat. UCP1 is used to regulate body temperature in mammals, while the function of AAC1-dependent proton transport is unknown. Activation of AAC1 and UCP1 is a powerful weight loss mechanism because the energy consumption it induces is not limited by the capacity of the muscles to use ATP. This makes it a powerful potential tool for addressing obesity, which is a serious public health problem [8]. However, existing AAC1/UCP1 activators are dangerous because they induce nonspecific currents across other biological membranes. Identifying the mechanism of AAC1 and UCP1-dependent proton transport would enable the development of AAC1/UCP1-specific uncouplers as safer weight loss drugs.

Proton transport by AAC1 is surprising because AAC1’s usual substrates, ATP and ADP, have charges of −3 to −4, and it is highly selective for these even over closely related AMP and guanine nucleotides [9,10]. The mechanism of proton transport remains a matter of debate, with conflicting evidence from different lines of work [4,11,12,13,14,15,16,17,18,19,20], and it remains unresolved whether AAC1 and UCP1 and different activators operate by the same mechanism. The sole activator-bound experimental structure—UCP1 bound to DNP—is nearly identical to the apo UCP1 structure and does not reveal the proton transport mechanism [21]. The residues at the binding site are not conserved in AAC1 [22], which is also activated by DNP [6], leaving unanswered how this site facilitates proton transport in UCP1 and raising the possibility that aspects of the mechanism may differ between AAC1 and UCP1. Furthermore, mutation of the UCP1 tryptophan W280 (PDB 8J1N numbering) that pi-stacks with the DNP in the experimental structure increased activation by the physiological activator AA [23,24], suggesting that AA may bind UCP1 at a different site or activate proton transport by a different mechanism than DNP. Structural details of the interactions between arachidonic acid and AAC1 and UCP1, as well as any AA-induced changes to the protein conformational ensemble, would therefore aid in identifying the mechanism of AAC1 and UCP1-dependent proton transport.

To date there is a single report of all-atom MD simulations of the recently determined UCP1 structures, and this work was performed in the presence of fatty acids [12]. Meanwhile there are two simulation studies of experimental AAC1 structures that have been performed in the presence of fatty acids [14,15], and two additional simulation studies of UCP1 in the presence of fatty acids that employed AlphaFold models [17,25]. In the former UCP1 study, negatively charged palmitoleic acid was placed in the IMS-solution and entered the central cavity, eventually binding between TM5 and TM6. In simulations of AAC1 by Bertholet et al., negatively charged AA from the IMS membrane leaflet also enter the central cavity and then bind in the TM5/TM6 groove (i.e., with its carboxylate in the cavity and its tail in the membrane) [15]. While starting with the FA in solution or in the membrane resulted in similar final positions, there is no guarantee that the sites accessible to aqueous FAs are the same as those for FAs initially in the membrane, where the concentration of FA is much higher (AA has a logP of 6.98 [26]). Arachidonic acid present at an equal concentration in the matrix leaflet of the membrane do not enter AAC1 in the Bertholet study [15]. However, Kreiter and co-workers [14] did observe a single negatively charged AA from the matrix leaflet slide along the AAC1-membrane interface and bind between TM2 and TM3. More recently, Jacobsen and colleagues [25] simulated a UCP1 AlphaFold model with neutral and negative lauric, palmitic, palmitoleic, linoleic, and stearic acid in both leaflets. They only observed negatively charged lauric and palmitoleic acid from the IMS leaflet enter the central cavity via the TM1/TM2 and TM5/TM6 grooves, while no FA entered from the matrix leaflet, consistent with the AAC1 results from Bertholet [15]. However, in simulations of another UCP1 AlphaFold model by Vojvodic et al. [17], with neutral and negatively charged arachidonic acid in both leaflets, one negative AA enters the TM4/TM5 groove from the matrix leaflet. Thus, while fatty acids in these simulations consistently bind to UCP1 and AAC1, where they bind and which leaflet they come from varies from study to study. This variation in results may arise from differences between AlphaFold models and experimental structures, fatty acid species, or other details of system construction or simulation parameters. To systematically compare the binding of AA to AAC1 and UCP1, we simulated the interactions of membrane AA with each protein under matching conditions and using experimental structures of each protein.

## 2. Results

### 2.1. Arachidonic Acid Binds in a Variety of Interhelical Sites and in the AAC1 IMS-Facing Cavity

**Figure 1:**
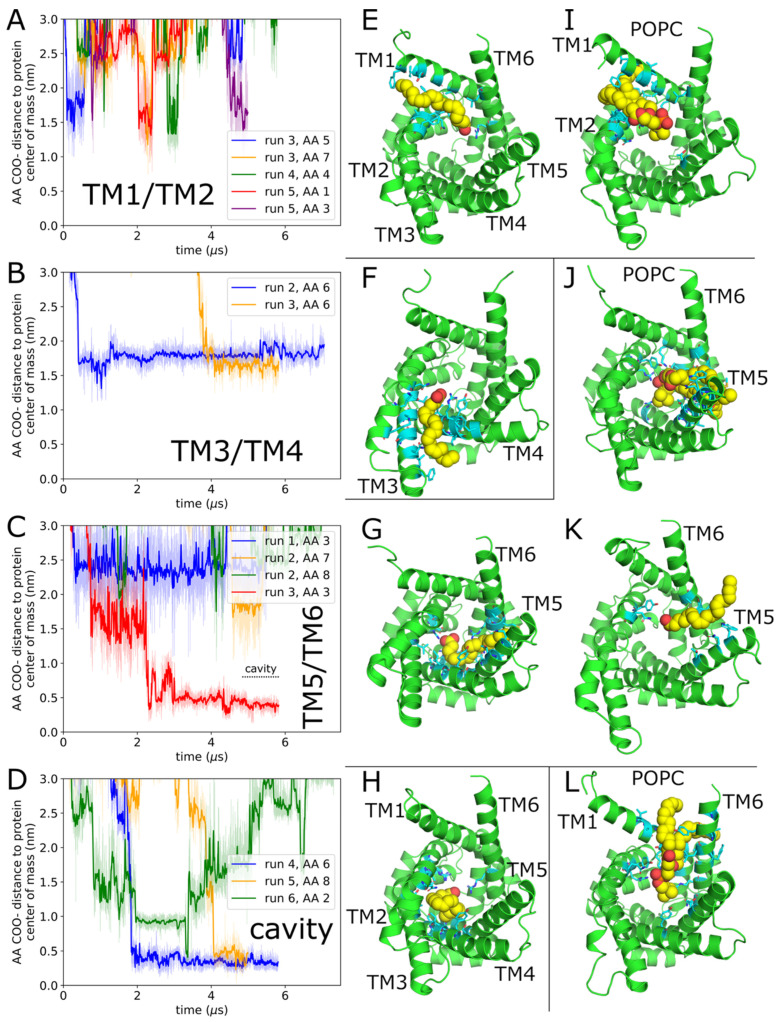
AAC1 binding sites. (**A**–**D**) Depth of the AA which bind in each site over time. In panel (**C**), the AA depicted by the red trace moves from TM5/TM6 to the IMS cavity over the last ~1 μs. The degree of independence of the two binding events of AA #6 to TM3/TM4 (panel **B**) and two AA #3 binding events to TM5/TM6 (panel **C**) is discussed in Methods. (**E**–**H**,**K**) Example snapshots of AA bound in the protein sites are shown in the plots to the left of each figure. In all molecular images, the protein is green, protein residues within 0.5 nm of AA are cyan, and AA is yellow (carbons) and red (oxygens). All images of the protein are oriented looking from the IMS (C) side. (**I**,**J**) Example snapshots of POPC bound in the protein sites highlighted in the plots to the left of each figure. Each POPC lipid is yellow (carbons), red (oxygens), and orange (phosphorus), and the protein color scheme is the same as for the AA bound images. (**L**) POPC bound in the TM6/TM1 groove, colored as above.

**Figure 2:**
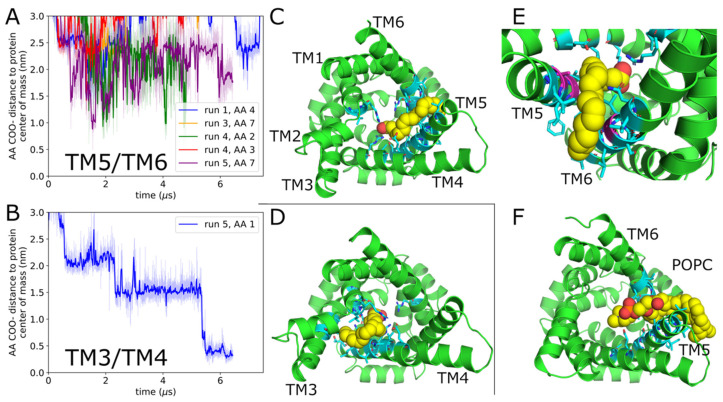
UCP1 binding sites. (**A**) Depth of the five AAs which bind in the TM5/TM6 groove over time. The degree of independence of the two binding events of AA #7 is discussed in Methods. (**B**) Depth of the AA which binds in the TM3/TM4 groove. (**C**,**E**,**F**) Example snapshots of AA (**C**,**E**) and POPC (**F**) bound in the TM5/TM6 groove. The protein is green, protein residues within 0.5 nm of the lipid are cyan, and AA/POPC are yellow (carbon), red (oxygen), and orange (POPC phosphate). Panel (**E**) is the closeup of the binding site depicted in panel (**C**), with Ala218, Gly222, and Gly278 shown in magenta rather than cyan. Panels (**C**,**F**) are oriented looking directly from the IMS (**C**) side, while panel (**E**) is also looking from the IMS side but at an angle to the membrane normal vector. (**D**) AA bound in the TM3/TM4 groove. The coloring scheme is as in panel (**C**), and the view is from the IMS side of the membrane.

Two simulation systems were constructed from AAC1 or UCP1, respectively, embedded in a 1-palmitoyl-2-oleoyl-sn-*glycero*-3-phosphocholine (POPC) bilayer with 8 AAs distributed randomly in each leaflet. Six parallel simulations of each system were run, with average lengths of 6 μs (detailed in Table 1). Simulations captured 14 deep AA-AAC1 binding events (Figure 1) and 6 deep AA-UCP1 binding events (Figure 2), as well as numerous POPC binding events, detailed in Table 2. Deep binding events are characterized by the AA carboxylate (or POPC phosphate) being in the central cavity within 1.3 nm of the protein center of mass, and (with the exception of the cavity bound states) the tail passing between two transmembrane helices and directly contacting both simultaneously (Figure 1E). In cavity bound states, the entire FA, including the end of the tail, is inside the central cavity (Figure 1H). We only observed binding events for AA or POPC from the C leaflet, and no AA or POPC lipids flipped from one leaflet to the other. Additionally, no AA or POPC bound between TM2/TM3 or TM4/TM5, probably because these helices are joined at their C ends by short loops, preventing them from separating to admit lipids or FA.

For the AAC1 simulations, most of the interhelical AA binding events are between helices TM1/TM2 or TM5/TM6. However, only one of the TM5/TM6 binding events (Figure 1G) is as deep as those reported previously for AAC1 [15]; a shallower event in the same groove is depicted in Figure 1K. In four cases, AA binds at the bottom of the IMS cavity. (Figure 1D and red trace in Figure 1C). All four events reached the cavity via the TM1/TM2 groove, in one case involving an intermediate TM5/TM6 binding event (Figure 1C red trace). AA bound between TM5/TM6, TM3/TM4, and in the central cavity exhibit multi-microsecond residence times, and in most cases, still reside there at the end of the simulation (Figure 1B–D), whereas those bound in the TM1/TM2 groove have residence times less than a microsecond (Figure 1A). POPC, like AA, binds most often in the TM1/TM2 and TM5/TM6 grooves (Figure 1I,J), but binds more deeply in the TM6/TM1 groove than AA (Figure 1L), and never binds deeply in the TM3/TM4 groove. Unsurprisingly given that POPC has two large hydrophobic tails and it does not have a net negative charge like AA, no POPC lipid fully extracted itself from the membrane into the central cavity.

For our UCP1 simulations, five of the six deep AA binding events occur between helices TM5/TM6 (Figure 2A,C,F), which is by far the largest interhelical groove in the cryo-EM structure [21]. In the three most deeply bound configurations, AA forms a salt bridge with Arg91. However, mutation of Arg91 (located on TM2 as shown in Figure 2C) has been reported to not affect proton transport [27], implying that this basic residue is nonessential for AA binding to the channel or potentially that proton uncoupling does not involve AA binding in the central cavity at the site reported here. To more directly test the relevance of the TM5/TM6 AA-bound UCP1 states observed in this study, smaller residues lining this site, such as Ala218, Gly222, and Gly278 (PDB 8HBV numbering; shown in magenta in Figure 2E), could be mutated to bulkier ones to occlude it. Jacobsen and colleagues also reported AA interactions with these latter two residues [12] as the protonated AA exited the TM5/TM6 groove to eventually reach the matrix leaflet. Given the size of the TM5/TM6 groove, it is not surprising that we also see POPC binding at a similar depth (Figure 2F), suggesting that AA binding may involve phospholipid displacement. The 6th deep AA binding event, which occurred between TM3/TM4, lasted for over 4 μs and was ongoing as of the end of the simulation, far exceeding the duration of any similarly deep TM5/TM6 binding event. The AA enters the interhelical groove in the first microsecond and moves progressively deeper into the C-state cavity in three discrete steps that each last at least a microsecond (Figure 2B). In the final configuration, the carboxylate is interacting with Arg83 and Arg182, and the tail is still between TM3/TM4 as depicted in (Figure 2D).

**Figure 3:**
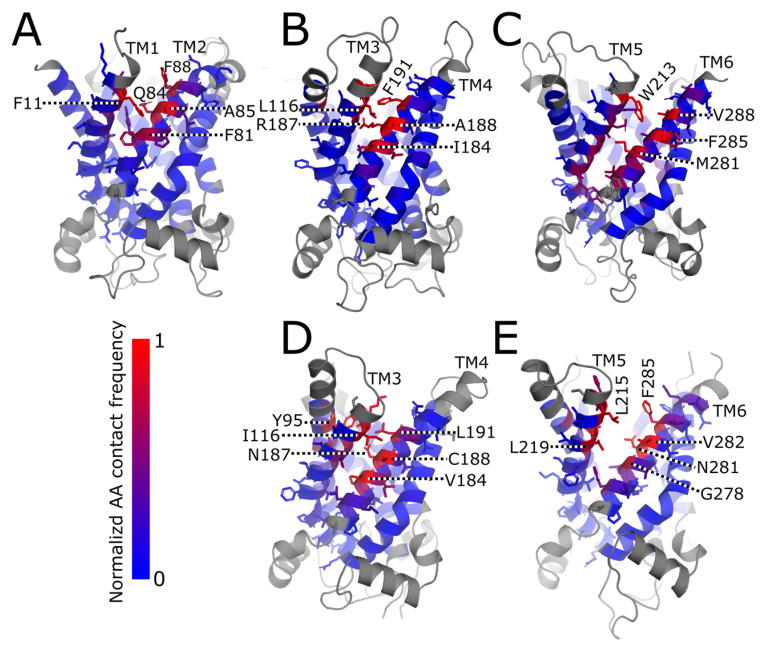
Residue-level contact frequencies for AA bound at each interhelical site. Contact frequencies are normalized by the overall frequency of AA binding at each site (i.e., a frequency of 1 means that the residue is always in contact with AA as long as there is an AA bound at the site). Residues are colored blue to red to indicate contact frequency, with residues which were omitted from contact frequency calculations in gray. Only the two helices lining the binding site are labeled in each panel and only sticks on these residues are shown (except for Y95 in panel (**D**) which is on TM2). Dotted black and white lines are used as arrows for labeling residues. (**A**–**C**) Three interhelical binding sites in AAC1. In panel (**B**), K91 on TM2 and N115 and G119 on TM3, which are unlabeled but shaded red, also have high contact frequencies. (**D**,**E**) Two interhelical binding sites in UCP1. In panel (**D**), K115 on TM3 is unlabeled but shaded red. In panel (**E**), A218 on TM5 similarly has a high contact frequency but is unlabeled.

The residues in AAC1 and UCP1 most frequently in contact with bound AA at each interhelical binding site were identified using a 0.5 nm heavy atom distance cutoff as described in Methods and are depicted in Figure 3. The residues with the most frequent AA contacts are mainly large hydrophobic residues such as Phe/Leu/Ile. Almost all of these residues are on the two helices lining the binding site and face inward towards it. The notable exception is Y95 on TM2 of UCP1 (Figure 3D), which is likely because TM2 buckles inwards in the simulation run in which an AA binds at the TM3/TM4 site. Only one or two binding events were observed at some sites, so the contact frequencies displayed here probably do not reflect the full ensemble of possible AA binding poses at each site.

### 2.2. Arachidonic Acid Is Enriched in the Membrane Adjacent to AAC1 and UCP1

**Figure 4:**
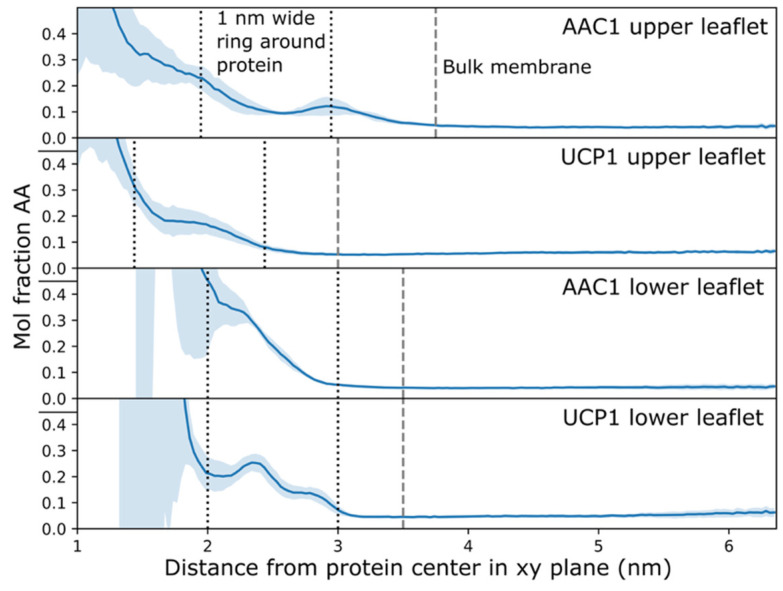
AA mole fractions around AAC1 and UCP1. Distances from the protein are computed using the coordinates of AA carboxylates and POPC phosphates. Shaded regions are the 1 standard deviation interval from six parallel simulations. Black dotted lines denote the membrane ring between 0 and 1 nm from the protein surface (see Methods for details). Gray dashed lines denote the beginning of bulk membrane, in which the lipid densities become constant as a function of radius.

In addition to examining binding of AA within AAC1 and UCP1, we attempted to determine if the fatty acid preferentially accumulated in the vicinity of the proteins. The mole fraction of AA ($mole\;fraction\;AA=\;\frac{AA}{AA+POPC}$) was calculated as a function of radius and averaged over time. Arachidonic acid is enriched in both leaflets of the membrane around AAC1 and UCP1 outside of deep binding sites in interhelical grooves (Figure 4). The region of the outer leaflet within 1 nm of the AAC1 surface (between dotted black lines) is enriched in AA roughly 3-fold compared to bulk (first panel). A corresponding 2-fold AA enrichment was observed in UCP1 (second panel). Meanwhile, the AA mole fraction enrichments relative to bulk for the same region in the inner leaflet are 4 and 3 -fold for AAC1 (third panel) and UCP1 (fourth panel), respectively. A 3–4 fold AA enrichment near AAC1 in a POPC membrane is consistent with the previous spectroscopic finding that there is a 4.1-fold ratio of stearic acid to POPC affinity for AAC1 [28]. AA enrichment could arise for several reasons, including better packing of its polyunsaturated tail against the protein than the monounsaturated tails of POPC, but we expect the major determinant is the preponderance of positive charge on these proteins which electrostatically attracts the negatively charged AA. We also expect that electrostatic effects explain the greater enrichments around AAC1 than around UCP1 because the former has a greater net charge of +19 while the latter only has a net charge of +11. Similarly, the greater enrichments in the inner leaflet likely arise from the larger number of positively charged basic residues there, particularly on the three short helices lying in the plane of the membrane.

### 2.3. Water Wires Form Through AAC1 and UCP1

**Figure 5:**
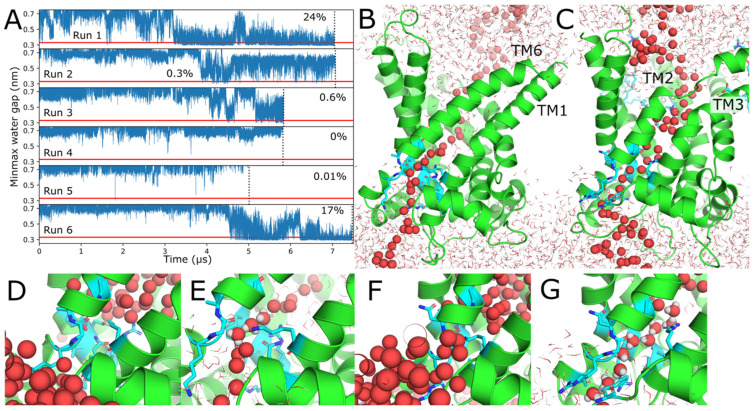
Water wires in AAC1. (**A**) Maximum water-to-water gap length along the path minimizing the maximum gap between C and M bulk water over time for each of the six simulation replicates. The plot for each replicate has the same y axis scale. The horizontal red line at 0.33 nm marks the threshold for water wire formation. The water wire formation frequency for each replicate is marked in the top right corner. (**B**,**C**) Water wires formed by the separation of TM6/TM1 or TM2/TM3. Residues lining the narrow portion of the water wire are shown in cyan. Also shown in cyan are AA and POPC present in interhelical grooves. Water molecules are shown as spheres or lines, with oxygen in red and hydrogen in white. Water molecules comprising the water wires are always shown in spheres, with hydrogens shown as spheres only for select molecules. All panels are oriented with the intermembrane space up and the matrix down. (**D**,**E**) Closeup views of the protein before and after formation of the water wire depicted in panel (**B**). (**F**,**G**) Closeup views of the protein before and after formation of the water wire depicted in panel (**C**). (**D**,**F**) depict the same starting state from different angles to match (**E**,**G**).

**Figure 6:**
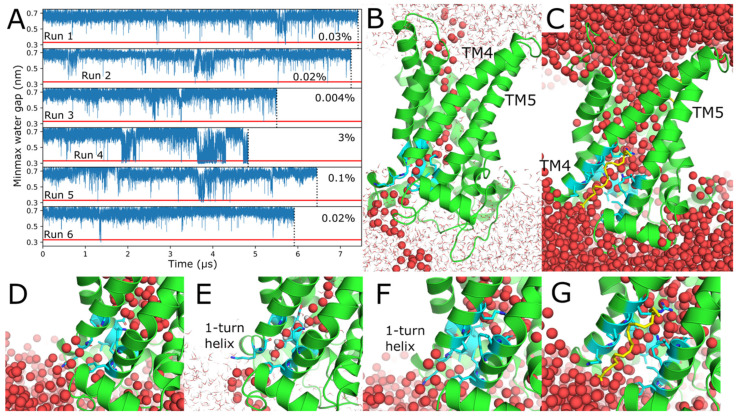
Water wires in UCP1. (**A**) Maximum water-to-water gap length along the path minimizing the maximum gap between C and M bulk water over time for each of the six simulation replicates. The plot for each replicate has the same y axis scale. The horizontal red line at 0.33 nm marks the threshold for water wire formation. The water wire formation frequency for each replicate is marked in the top right corner. (**B**,**C**) Water wires formed by the separation of TM4 and TM5. Residues lining the narrow portion of the water wire are shown in cyan. Water molecules are shown as spheres or lines, with oxygen in red and hydrogen in white. Water molecules comprising the water wires are always shown in spheres, with hydrogens shown as spheres only for select molecules. An arachidonic acid in the membrane next to the water wire in panels C and G is shown in yellow. All panels are oriented with the intermembrane space up and the matrix down. (**D**,**E**) Closeup views of the protein before and after formation of the water wire depicted in panel (**B**). (**F**,**G**) Closeup views of the protein before and after formation of the water wire depicted in panel (**C**). (**D**,**F**) depict the same starting state from slightly different angles and with different residues in cyan to match E and G.

The C-state structures of AAC1 [22,29] and UCP1 [21,30] show that the bottom of the cavity is sealed off from the M side by well-packed protein residues. There is no obvious series of protonatable side chains through this insulating gap that could serve as a conduit for protons to move from the IMS to the matrix. Some models of proton transport therefore postulate a conformational change to a conducting state in which the gap is bridged by waters and/or protonatable residues, which could conduct protons via Grotthuss shuttling [31]. However, no such state has been observed, and previous MD studies have reported the protein to be water-impermeable [17,25]. Our simulations were therefore examined for the presence of water wires, which we defined as series of water molecules connecting bulk water on the C and M sides of the protein with a maximum distance of no more than 0.33 nm between consecutive oxygens (marked by the horizontal red lines in Figure 5A and Figure 6A). Water wires were observed in five out of six AAC1 simulations and all six UCP1 simulations. Water wire formation frequencies (listed on the right of Figure 5A and Figure 6A) varied widely between simulations, with some replicates entering states where water wires formed regularly and others not doing so. In no case were water wires present continually for periods longer than 12 ns, as can be seen from the flicker of the minimum gap distance across the horizontal dashed line in all panels. Thus, unlike proteins like SGLTs, sugar transporters that leak water via multiple pathways with larger open pores that remain persistently open and solvated [32], UCP1 and AAC1 are constantly reforming their insulating gap or de-wetting even during periods of frequent water wire formation. Water wire lifetime distributions are detailed in Figure S1, with longer lived wires being roughly exponentially less likely.

Water wires in AAC1 formed through all three pseudosymmetric units of the protein (TM2/TM3, TM4/TM5, and TM6/TM1). The average water wire formation frequency across all runs was 7%, but due to the wide variation in water wire formation frequency between simulation runs, the standard deviation of the formation frequencies exceeds the mean, so this average formation frequency is not a reliable estimate of the true formation frequency. The water wires formed between TM2/TM3 42% of the time (panel C), 2% formed between TM4/TM5, and 56% formed between TM6/TM1, with multiple units forming wires roughly concurrently in some cases (Figure S2A). In the 1st and 6th runs, water wires form frequently starting around 3 and 4.5 μs, respectively, and a less dramatic but similarly discrete increase in frequency occurs in the 2nd and 3rd runs (Figure 5A). The width and geometry of the wires varied, as illustrated by the variable length of the constricted region in Figure 5 panels E and G, and the alternative wire path in Figure S2B.

Water wires formed through UCP1 an average of 0.5% of the time, but as in AAC1 the frequency varies widely between runs. Unlike AAC1, all water wires through UCP1 formed between TM4/TM5 as depicted in Figure 6 panels B-G. In the 4th run, there are three distinct periods around 2, 4, and 4.7 μs during which water wires form frequently (Figure 6A), while in all other UCP1 simulations water wire formation is far less common and consists of isolated events. The water wire depicted in Figure 6B,D,E features a constriction with a single file line of waters with aligned hydrogen bonds compatible with Grotthuss shuttling [33], while the one depicted in panels C and G is wider. The single-turn alpha helix just past the matrix end of TM4 in Figure 6B,D–F joins with TM4 to form a continuous helix in panels C and G. This conformational change, which occurs during the period of frequent water wire formation in run 4, may stabilize the formation of a water wire. It should be noted that the cardiolipin bound near the M ends of TM4 and TM5 is dissociated from the protein in the state depicted in C.

To more closely probe whether water wire formation seen in this work might be a result of local or global unfolding, we calculated the C-alpha RMSD and root mean square fluctuations (RMSF) of the entire protein to the experimental structure, including the modeled loops that are missing in the deposited structures. As shown in Figure S3A, based on qualitative, visual inspection, UCP1 RMSFs were slightly smaller than those reported by the Pohl lab [17], and similar to those reported by the Khandelia lab [25], both of which used AlphaFold models and report the protein to be water-impermeable. However, the RMSD values are much lower when starting from the solved structure of UCP1 (PDB ID 8HBV), which yields values around 0.2 nm (Figure S3B), compared to values around 0.4 nm reported for the above two studies using AlphaFold structures. Gagelin et al. [24] also report low levels of water permeability from simulations of the experimental AAC1 structure and UPC1 AlphaFold models, though they do not report on the existence of fully connected water wires nor report whole-protein RMSDs for comparison. Furthermore, comparison of RMSD values in simulation frames with and without water wires found no statistically significant increase in RMSD upon water wire formation in UCP1, and a statistically significant increase of only 0.06 nm in AAC1 (Figure S4), counter to the notion that water wire formation reflects large scale deviation from the experimental structure.

It has been proposed by Klingenberg [4] and our lab together with the Kirichok lab [15] that AA induces a conformational change in UCP1 and AAC1, which enables proton permeation in addition to its ability to directly bind and stabilize protons in the cavity. Both the depth of the deepest-bound AA and the frequency of water wire formation typically increased over time throughout our simulations, indicating that we had yet to reach the equilibrium ensemble of either observable. We were unable to identify a correlation between AA binding and water wire formation independent of this increase in time. Moreover, the relationship between the two variables varied widely between simulations, as depicted in Figure S5A. The same was also true for the relationship between AA binding and protein RMSD, shown in Figure S5B.

We also investigated whether water wire formation might be a result of dissociation of the three bound cardiolipins [34], which are partially resolved in experimental structures. These cardiolipins are known to be important for AAC1 ATP/ADP transport [10] and UCP1 stability [35] and were included in our simulations as well as those by Pohl and Khandelia. Cardiolipins moved to locations more than 3 nm from the protein center of mass in the plane of the membrane (having started just under 2 nm away) in four cases in UCP1, and transiently in one case in AAC1, but in most cases they subsequently re-bound, and in no case did multiple cardiolipins dissociate at once (Figure S6A). No statistically significant correlation was observed (at alpha = 0.05) between the distance of the furthest cardiolipin from the protein center of mass in the plane of the membrane and the formation of water wires (Figure S6B). The one UCP1 simulation in which water wires formed regularly (run 4, depicted in Figure 6C) also features cardiolipin dissociation, but the temporal correlation is far from perfect and water wires form regularly around 2 μs while cardiolipin remains bound.

**Figure 7:**
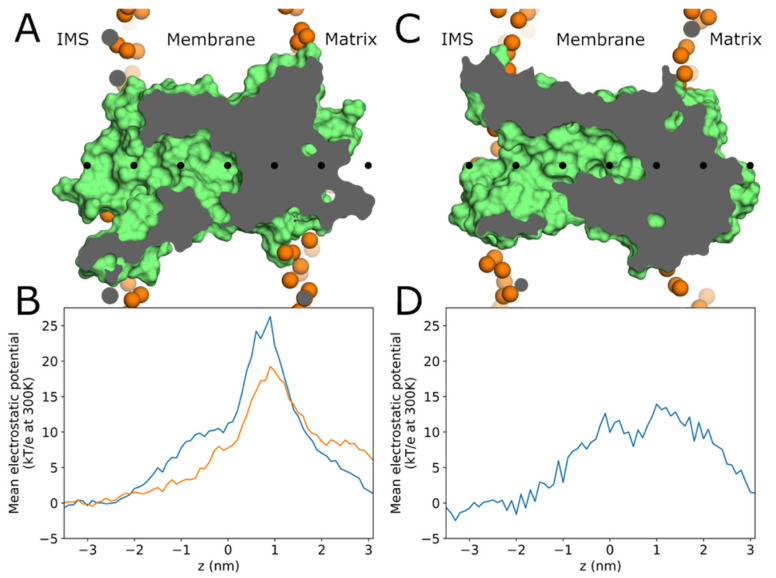
Electrostatic potential landscape along water wires. (**A**) Cross section of equilibrated AAC1 (green surface image), showing the insulating gap (dark gray interior of the protein). Black dots mark the center of mass and points along the z axis at 1 nm increments. (**B**) Average electrostatic potentials along water wires through AAC1 as a function of z (relative to the protein center of mass z coordinate) in simulations 1 (blue) and 6 (orange), with the z (horizontal) axis aligned to match panel (**A**). Electrostatic calculations were performed using VMD PMEPot plugin [37,38] as discussed in the Methods. (**C**) Cross section of equilibrated UCP1, again showing the insulating gap as in panel (**A**). (**D**) Average electric potentials along water wires through UCP1 as a function of z in simulation 4, with the z (horizontal) axis scaled and shifted to match panel (**C**).

Previous studies have reported a large positive electric potential of approximately 1 Volt in the insulating gap of AAC1 [36] and UCP1 [17], but the reported potential varies in different parts of the gap. Such a potential would repel protons, inhibiting proton conduction via the water wires shown in Figure 5 and Figure 6. To obtain a more precise understanding of the electric potential along the water wires observed in our simulations, we estimated the electrostatic potential at the coordinates of the water wire water oxygen atoms using the VMD PMEPot plugin [37,38]. These potentials were averaged across multiple wires (every 10th wire-containing frame for selected runs) to obtain an estimate of the typical electric potential landscape along water wires, as depicted in Figure 7. The potential (relative to bulk water) in AAC1 rises to 8 kT/e (orange curve for run 6) and 10 kT/e (blue curve for run 1) at the center of the protein (z = 0) near the bottom of the IMS cavity (Figure 7A,B), consistent with continuum electrostatics calculations of the potential along the protein’s z axis in our lab’s previous work on AAC1 [15]. The potential then rises an additional 10–15 kT/e in the middle of the insulating gap to 19–26 kT/e above bulk, equivalent to 0.74–1 V (again for runs 6 and 1, respectively). Variation in the shape and height of the barrier between AAC1 runs 1 and 6 indicate that the barrier geometry is sensitive to the details of the protein and water wire configuration. Longer simulations or enhanced sampling may therefore be necessary to accurately capture the full ensemble of electrostatic barrier heights. The electrostatic potential barrier in UCP1 reached a much lower height of 0.54 V (Figure 7C,D) and peaked in the insulating gap as in AAC1. The higher average barrier in AAC1 than in UCP1 is likely attributable to the former’s greater net charge (+19 vs. +11). However, the maximum potential along each individual water wire averages 34 kT/e in AAC1 and 31 kT/e in UCP1, which exceeds the maximum of this profile, because different water wires reach their maximum electrostatic potential at different z coordinates, as shown in Figure S7. For a mitochondrion with a −160 mV transmembrane potential, half of which had been traversed at the midpoint of the insulating gap, the potential in the insulating gap would be lowered by a mere 80 mV, or 3 kT for H^+^. AA molecules do not enter the insulating gap in our simulations, and so they could not directly stabilize protons there by becoming protonated and would be unlikely to significantly reduce the potential there with their negative carboxylates.

## 3. Discussion

The deep interhelical binding sites observed in our MD simulations provide a potential explanation for a variety of experimental observations, suggesting that these states are functionally relevant to AA-activated proton transport. AA in this work are only observed to bind at interhelical sites from the IMS-facing membrane leaflet, consistent with previous findings from our lab in AAC1 simulations initiated from both C and M-states [15]. Electrophysiology experiments on mitochondrial membranes in which all UCP1 are oriented in the same direction correspondingly report that AA must be added from the IMS side of the membrane in order to induce a proton current [23]. POPC binds in most of the same interhelical sites as AA, suggesting that POPC-like molecules with greater affinity could act as competitive inhibitors of AA. One report potentially supports this hypothesis, as lysophosphatidic acid, which combines the acyl-glycerol-phosphate structure of POPC (albeit with one tail) with the negative charge of AA, is an inhibitor of AA-induced proton current [23]. Finally, while no co-structures have been determined that reveal a FA or PC-lipid with UCP1 or AAC1, the TM5/TM6 groove in the experimental apo structure of UCP1 (PDB 8HBV) solved in nanodisc contains unmodeled electron density consistent with the presence of a lipid. The glycerol and upper carbons of the FA tails of POPC bound in this groove in our simulations in poses roughly matching this density, and future structural studies supplemented with brominated lipid probes to provide higher contrast [39] or with AA or other FAs may verify the binding sites predicted here.

With the exception of 4 instances where AA left the bilayer and resided entirely in the AAC1 cavity, deeply bound AAs have their tails buried in the hydrophobic core of the membrane, and their carboxylate group and the first few carbons of the AA in the water-filled IMS-facing cavity. Structure activity relationship (SAR) studies of fatty acid activators of UCP1 generally report that the addition of hydrophilic groups on the second FA carbon (i.e., 2-hydroxylauric acid, 2-hydroxymyristic acid, and 2-hydroxypalmitic acid) preserve most, and in some cases all, of the activator-induced inhibitor-sensitive proton current, while addition of such groups at the last carbon (i.e., in 12-hydroxylauric acid, tetradecanedioic acid, hexadecanedioic acid, and 16-hydroxypalmitic acid) eliminates most proton current [40,41]. The former set of lipid modifications add hydrophilic groups near the negatively charged carboxylate, which is always situated in the water-filled IMS cavity in the bound states we observe, while the second modification adds a strong polar character to the tails which would be unfavorably buried in the hydrophobic core of the membrane, destabilizing the interhelical bound state. Thus, our simulations suggest that AA adopts similar conformations in UCP1 and AAC1 in which the polar headgroup is in the water filled cavity and the tails are in the bilayer consistent with this SAR data [40,41].

The unsaturated double bonds on the AA tails of AAC1- and UCP1-bound structures typically lie in the constricted region between the TM helices when AAs are deeply bound to the protein. A more saturated FA would lose rotational degrees of freedom there and pay an entropic price. AA has fewer such degrees of freedom to lose owing to the nonrotatable nature of its four double bonds and should therefore have a lower entropic penalty to binding. This effect is however difficult to disentangle from a related fact that unsaturated FA also pack less well with the more saturated POPC tails than more saturated lipids, lowering the cost of leaving the bulk membrane to bind to the protein even in the absence of binding-site-specific features. Additionally, the double bonds have a slightly higher polar character, which may benefit from interactions with charged groups on the protein compared to the more non-polar character of the bilayer core. All three of these effects potentially explain the greater potency of more saturated FA as UCP1 activators, though other explanations for this have been put forward on the basis of proton transport mechanisms not dependent upon the sorts of interhelical binding sites reported here [7].

This study reports AA interactions with AAC1 and UCP1 in a zwitterionic POPC membrane. However, a quarter of the IMM is composed of negatively charged cardiolipins [42,43], which would likely compete with AA for binding to the surface of the protein. The AA enrichments reported here therefore likely overestimate the true enrichment in the IMM. The physiological AA distribution may be further complicated by the presence of other non-POPC lipids and cholesterol, as well as the asymmetry between the lipid composition of the inner and outer IMM leaflets. FA enrichment around the protein has nevertheless been reported in simulations of UCP1 AlphaFold models with shorter and less saturated FA in cardiolipin-containing membranes [25]. Bertholet et al. ran simulations of AAC1 in both membranes composed of POPC only and membranes composed of a mixture of phosphatidylcholine, phosphatidylethanolamine, and cardiolipins, and reported that membrane composition had no effect on AAC1 structure or ligand binding [15]. Furthermore, FA-induced AAC1-dependent proton transport has been demonstrated experimentally in liposomes reconstituted from a lipid mixture containing only 3.6% cardiolipin by weight [1]. FA-induced UCP1-dependent proton transport has similarly been demonstrated in liposomes reconstituted with 1% cardiolipin [5]. These results imply that the AAC1 and UCP1-FA interactions needed for FA-induced proton transport do not require the high cardiolipin content of the inner mitochondrial membrane. AA and AAC1/UCP1-dependent proton transport has also been reproduced in planar bilayers [6,7], indicating that membrane curvature is not required for the proton transport mechanism. The planar POPC-based simulation systems in this study should therefore be capable of identifying key protein-FA interactions involved in proton transport.

Electrostatics calculations using VMD’s PMEPot plugin by both our lab and the labs of Pohl [17] and Tajkhorshid [36] identified electric potentials sufficient to inhibit water-wire-dependent proton conduction through AAC1 and UCP1. However, PMEPot computes only the reciprocal component of the PME potential, omitting short range terms which would create infinities in grid-based potential calculations [37,38] but would have large effects on the potential experienced by protons. Furthermore, the maximum potentials along different water wires in this study had a standard deviation of 8 kT/e, making the single average potential unsuitable for rate calculations since rates vary exponentially with transition state energies. Electrostatic calculations also do not capture the desolvation energy or any other components of the energy of a proton traversing a water wire, nor do they consider the effect of the excess proton itself on water wire formation [44]. Future studies using reactive MD to explicitly model excess protons could capture the full energy landscape of water wire traversal by protons and would explicitly account for variations in potential between different wire configurations. Moreover, it would be informative to perform classic MD using a more accurate 4-point water model such as OPC water [45], combined with a force field such as Amber19SB [46], as this would likely yield a more accurate picture of the energy landscape of water wire formation.

## 4. Methods

### 4.1. Simulations

#### 4.1.1. System Construction

*Protein And Cardiolipins*. A model of apo AAC1 with three bound cardiolipins (derived from PDB ID 2C3E chain A) was downloaded from the Zenodo repository containing computational materials from reference [15]. This 2C3E-based model was used because bovine AAC1 structures have better sequence homology to human AAC1 (of which there is no published structure) than fungal ones, and 2C3E was chosen over the other bovine structure (1OKC) for consistency with our previous work. A cryo-EM structure of apo-UCP1 (PDB ID 8HBV chain A) [21] was downloaded from RCSB. All molecules other than the protein were removed. Unmodeled side chains and the C terminal carboxylate were added manually in PyMOL 3.0.0 [47]. Three tetralinoleoylcardiolipin molecules were modeled manually in PyMOL around UCP1, with phosphates positioned at the N termini of TM2, TM4, TM6, and the three shorter M side linker helices in positions consistent with those of lipids from the UCP1 cryo-EM structures, which contained no fully resolved cardiolipin molecules.

*CharmmGUI*. Each protein-cardiolipin system prepared above was solvated and embedded in an arachidonic-acid-containing POPC membrane using CharmmGUI membrane builder [48,49,50] with a composition described in Table 3. Arachidonic acid and POPC were randomly distributed in the membrane. The CharmmGUI KCl concentration was set to 150 mM with ion numbers set to yield a zero net charge for the whole system. Protonation states were assigned based on a pH of 7 (positive arginine and lysine, negative aspartate and glutamate, charged protein termini, all other protein residues neutral), and histidines were constructed with the hydrogen attached to the π-nitrogen (the one nearer to the backbone). The membrane composition yielded average box x and y lengths of 9.2 nm in production MD. The system was parameterized with the Charmm36m [51] force field for the protein, Charmm36 [52] for lipids and AA, TIP3P water [53], and the default Charmmgui ion parameters [54].

#### 4.1.2. MD Simulations

*General*. Simulations 1–4 for each protein were performed with Gromacs 2024.4, while simulations 5 and 6 were performed with Gromacs 2024 [55]. Non-water hydrogen atoms were constrained using LINCS [56]. Long range electrostatic forces were calculated with Particle Mesh Ewald (PME) with a Fourier spacing of 0.12 [57]. Van der Waals forces were switched off smoothly between 1 nm and 1.2 nm. Thermostats were set to 310.15 K. NPT simulations were performed with a semiisotropic barostat at atmospheric pressure (1.01325 bar) and a compressibility of 4.5 × 10^−5^ bar^−1^.

*Equilibration*. Each system was energy-minimized with harmonic restraints using the steepest descents algorithm, equilibrated in an NVT ensemble with harmonic restraints for 0.1 ns, an NPT ensemble for a total of 40.3 ns as the restraints were gradually removed, and then equilibrated for an additional 20 ns without restraints. The equilibration protocol is detailed in Table 4. A 2 fs timestep was used for all dynamics steps.

*Production MD*. Each equilibrated system was used as the starting configuration for six parallel simulations with the v-rescale thermostat and c-rescale barostat. The thermostat and barostat are stochastic, and random seeds were used. Configurations were saved every 50,000 steps. Simulation lengths and timesteps are given in Table 1. Hydrogen mass repartitioning (implemented using the Gromacs 2024 mass-repartitioning-factor option) was used for systems with 4 fs timesteps.

### 4.2. Analysis

*Software*. Trajectories were viewed using VMD version 1.9.4a55 [61] and PyMOL versions 3.0.0 (linux) and 3.1.6.1 (mac) [47]. Analysis was performed in python using MDTraj version 1.10.0 [62]. The PMEPot VMD plugin was used to estimate electric potentials [37,38] with the default settings. ChimeraX version 1.10.1 was used to inspect electron densities from experimental structures [63]. ChatGPT 5.0 was used to write a file format conversion script, and the autocompletion function of the Visual Studio Code GitHub copilot extension v1.219.0 provided general programming assistance.

*AA contact frequency calculation*. For each interhelical binding site (i.e., consecutive pair of transmembrane helices), the binding state (bound or unbound) of each AA in each frame of each simulation was defined as follows: The AA was considered to be bound if its tail intersected a surface * spanning the gap between the helices and was in contact (heavy atom distance < 0.5 nm) with atoms of residues from both helices. For each site, the binding frequency for each residue was defined as the fraction of frames in which the AA was bound at that site in which it was also in contact with an atom from that residue.

* Composed of two triangles defined between the transmembrane helices (the residues used to define these triangles can be found in the github linked below).

*AA mol fraction calculation*. The surface of the protein in the outer leaflet is defined by the radius of the farthest ends of the transmembrane helices on the C side of the protein. In the inner leaflet a radius of 2 nm was used for both proteins to match the exterior radius. In each case the protein was approximated as a cylinder.

*Water wire gap calculation*. Water molecules in a cylinder centered on the protein and extending into bulk water on either side, with its long axis perpendicular to the membrane, were selected for further analysis. The distance matrix of the oxygen atoms of those water molecules (and any AA carboxylate oxygens in the cylinder) was calculated (with distances above 1 nm omitted for computational efficiency as they are irrelevant for this work) and used to construct a minimum spanning tree. A water molecule in bulk water near either end of the cylinder was selected, and the path between them through the minimum spanning tree was identified. The length of the longest edge along this path is the maximum gap along the water wire. This length represents the longest jump a proton being conducted between the endpoint waters by Grotthuss shuttling would need to make. It therefore serves as a proxy for potential proton conduction rate, though it lacks the quantitative precision of the method described by Li and Voth [44]. This minimum-spanning-tree based method, which is to our knowledge novel, benefits from its lack of tunable parameters and its physically interpretable protein-agnostic output in terms of an intermolecular distance.

*Trajectory independence*. To examine the independence of AA trajectories, we calculated the distances between carboxylates of AA replicates which had begun at the same position in each pair of simulations (i.e., the distance between AA number 4 in AAC1 run 2 and AA number 4 in AAC1 run 3), using trajectories in which the protein was centered. At some point between 0 and 2 microseconds, every pair of AA replicates in AAC1 had reached a distance of at least 4.8 nm apart, and all but two pairs of AA replicates in UCP1 had reached a distance of at least 5 nm apart. The two pairs of UCP1 AA replicates which failed to diverge to 5 nm in the first two microseconds reached 3.8 and 4.7 nm, respectively, and were both replicates of the same AA, which was located in the M leaflet. The divergence of the positions of essentially every pair of AA replicates early in the simulation indicates that their trajectories are largely independent in spite of their identical starting configurations. An example of this divergence for two replicates of an AA which later bind at the same site on the protein is shown in Figure S8.

*Script availability*. Scripts used for analysis, along with simulation input and .mdp files, are available on GitHub at: https://github.com/JonathanHB/arachidonic_acid_aac1_ucp1_interactions.

## Figures and Tables

**Table 1:** List of simulations.

Protein	Run	Timestep (fs)	Save Frequency (ps)	Steps	Time (μs)
AAC1	1	4	200	35,242	7.0484
AAC1	2	4	200	35,336	7.0672
AAC1	3	2	100	58,325	5.8325
AAC1	4	2	100	58,169	5.8169
AAC1	5	4	200	25,043	5.0086
AAC1	6	4	200	37,351	7.4702
UCP1	1	4	200	37,084	7.4168
UCP1	2	4	200	36,265	7.253
UCP1	3	2	100	55,026	5.5026
UCP1	4	2	100	48,309	4.8309
UCP1	5	4	200	32,256	6.4512
UCP1	6	4	200	29,577	5.9154

**Table 2:** Deepest binding states of AA and POPC.

	AA		POPC	
Site	AAC1	UCP1	AAC1	UCP1
TM1/TM2	5	0	8 *	0
TM3/TM4	2 *	1	0	0
TM5/TM6	4 *	5 *	6	7 **
TM6/TM1	0	0	4	0
IMS cavity	3	0	0	0

* Same FA in two parallel simulations. The degree of independence of these observations is discussed in Methods. ** Same FA in four parallel simulations.

**Table 3:** System compositions.

Molecule	Number in AAC1 System	Number in UCP1 System
Protein (AAC1 or UCP1, respectively)	1	1
Tetralinoleoylcardiolipin (TLCL2; charge−2)	3	3
1-palmitoyl-2-oleoyl-sn-*glycero*-3-phosphocholine (POPC)	211 (107 in M leaflet, 104 in C leaflet)	217 (107 in M leaflet, 110 in C leaflet)
Arachidonic acid (ARAN; charge−1)	8 per leaflet (16 total)	8 per leaflet (16 total)
Water	14,375	14,595
Potassium (K+)	41	49
Chloride (Cl−)	38	38

**Table 4:** Equilibration parameters.

Step	Length (ns)	Protein Backbone Restraints	Protein Sidechain Restraints	Restraints on All Other Molecules	Thermostat	Barostat
1 (minimization; no dynamics)	n/a; up to 500,000 steps; emtol = 1000 kJ·mol^−1^·nm^−1^	4000	4000	400	n/a	n/a
2 (NVT)	0.1	4000	4000	400	Berendsen [58]	none
3	0.1	4000	4000	400	Berendsen	Berendsen
4	0.1	2000	2000	80	Berendsen	Berendsen
5	0.1	2000	2000	16	Berendsen	Berendsen
6	20	2000	2000	0	Berendsen	Berendsen
7	2	2000	1000	0	Berendsen	Berendsen
8	2	2000	400	0	Berendsen	Berendsen
9	2	1000	400	0	Berendsen	Berendsen
10	2	400	200	0	Berendsen	Berendsen
11	2	200	20	0	Berendsen	Berendsen
12	10	20	0	0	Berendsen	Berendsen
13	20	0	0	0	v-rescale [59]	c-rescale [60]

Distance force constants have units of kJ/(mol·nm^2^).

## Data Availability

The data presented in this study are openly available in GitHub at: https://github.com/JonathanHB/arachidonic_acid_aac1_ucp1_interactions.

## Supplementary Materials

Supporting information can be downloaded at: https://www.mdpi.com/article/10.3390/ijms262110504/s1.
