# Supplementary material for: Interactions of Arachidonic Acid with AAC1 and UCP1
Source: Int J Mol Sci. 2025 Oct 29;26(21):10504. doi: 10.3390/ijms262110504 (PMC12607696; doi:10.3390/ijms262110504)

Supplementary information for *Interactions of Arachidonic acid with AAC1 and UCP1*

Jonathan Borowsky and Michael Grabe*

Cardiovascular Research Institute, Department of Pharmaceutical Chemistry, University of California, San Francisco, San Francisco, CA 94158, USA; jonathan.borowsky@ucsf.edu

* Correspondence: michael.grabe@ucsf.edu

Figure S1. Water wire lifetime distributions.

A. Water wire lifetime distribution for AAC1, with 5549 wire formation events.

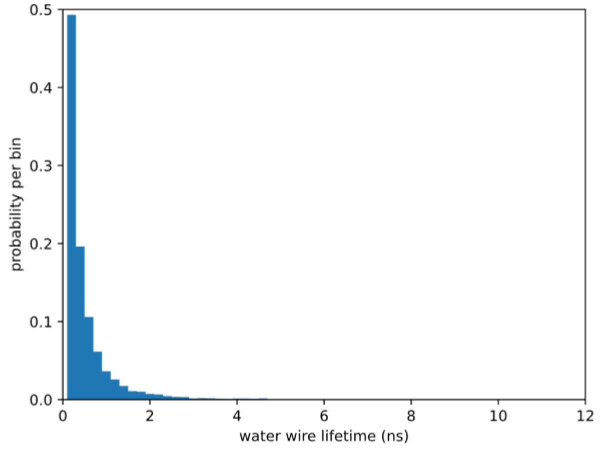

B. Water wire lifetime distribution for UCP1, with 856 wire formation events.

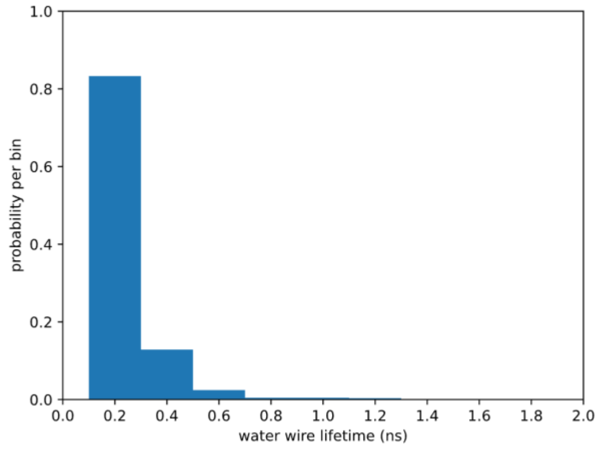

Figure S2. Specific water wire configurations in AAC1.

A. Two simultaneous water wires through AAC1, between TM1/TM6 (left wire) and TM2/TM3 (right wire), shown in orange.

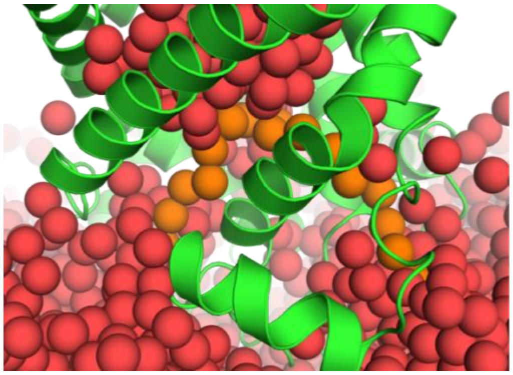

B. A water wire running along the outside of AAC1 between TM5 (left helix) and TM6 (right helix).

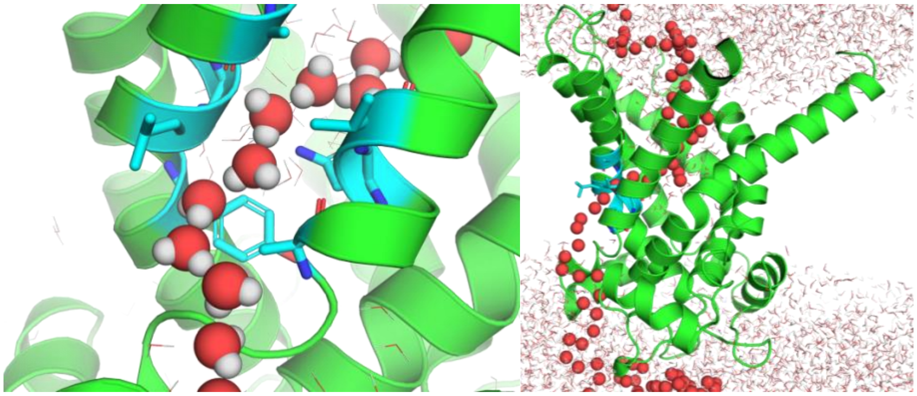

Figure S3. RMSF and RMSD plots for AAC1 and UCP1 simulations.

A. RMSFs of AAC1 and UCP1

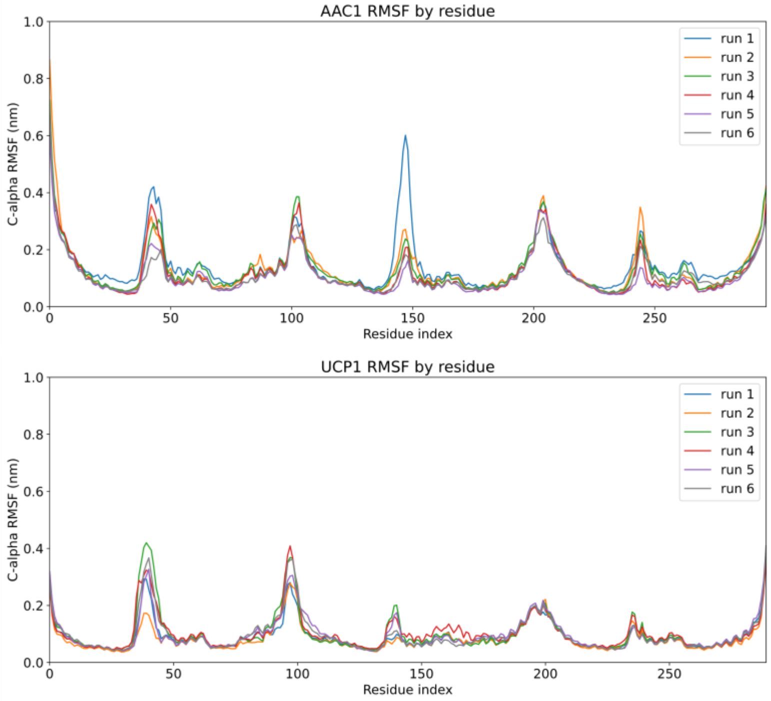

B. RMSDs of AAC1 and UCP1

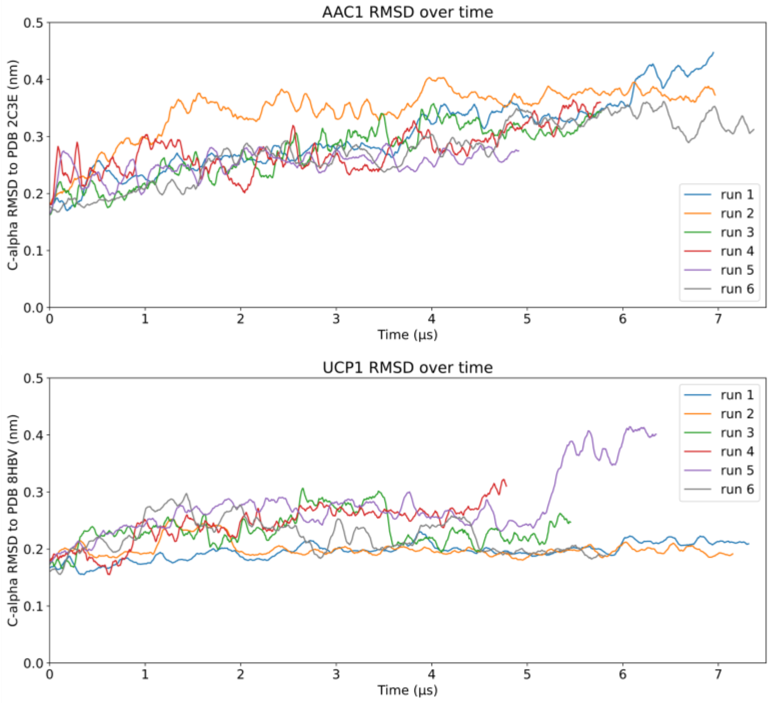

Figure S4. RMSD changes upon water wire formation in each simulation. 95% CI for AAC1 is (0.02 nm, 0.08 nm), and 95% CI for UCP1 is (-0.007 nm, 0.05 nm). Data points for the different runs are colored as in Fig. S3.

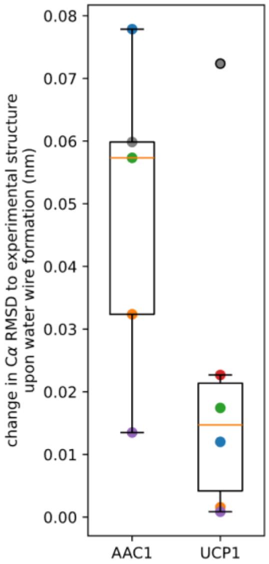

Figure S5. AA binding versus water wire formation (panels in A) or RMSD (panels in B). Each data point represents one simulation frame. Data points are colored purple to yellow as time progresses.

A. AA binding vs water wire formation. Each panel has the same vertical scale.

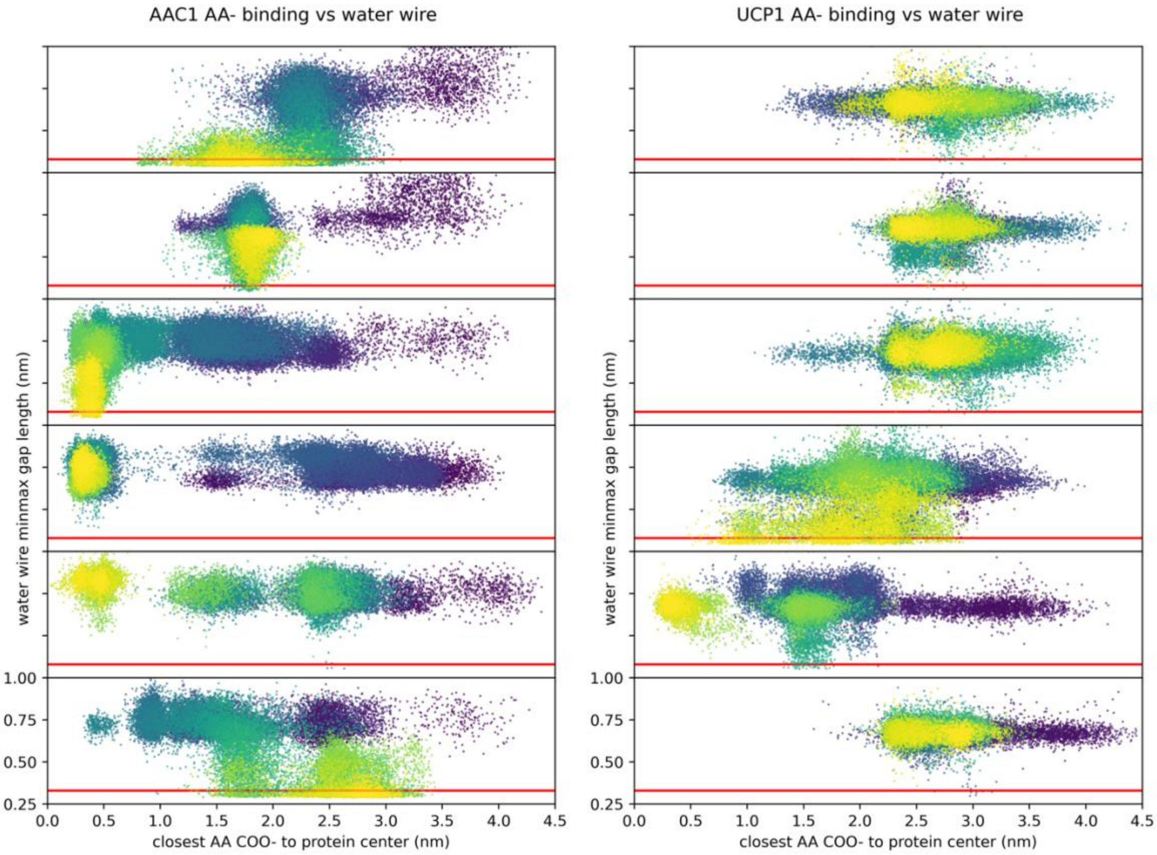

B. AA binding vs RMSD. Each panel has the same vertical scale.

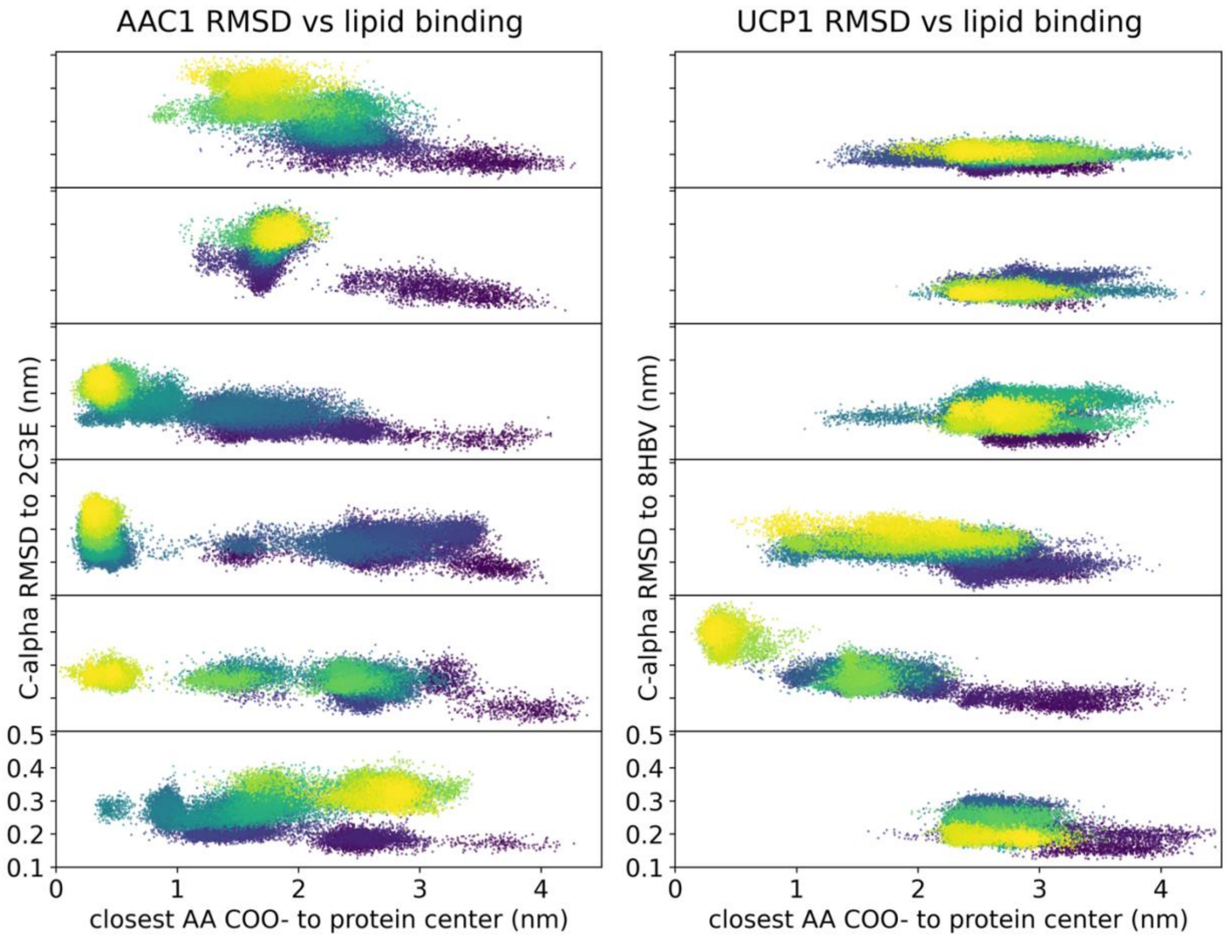

Figure S6. Analysis of cardiolipin lipids.

A. Cardiolipin distances to protein center of mass in the xy plane (the plane of the membrane) over time, measured from the central carbon of the cardiolipin's central glycerol. Each cardiolipin began bound at or near the ends of two transmembrane helices, and is labeled with the names of those helices.

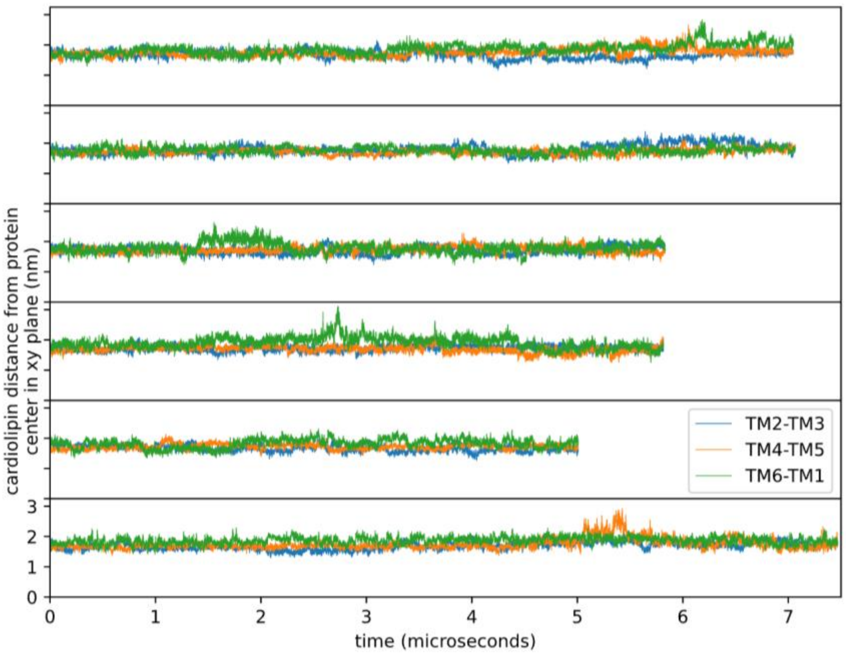

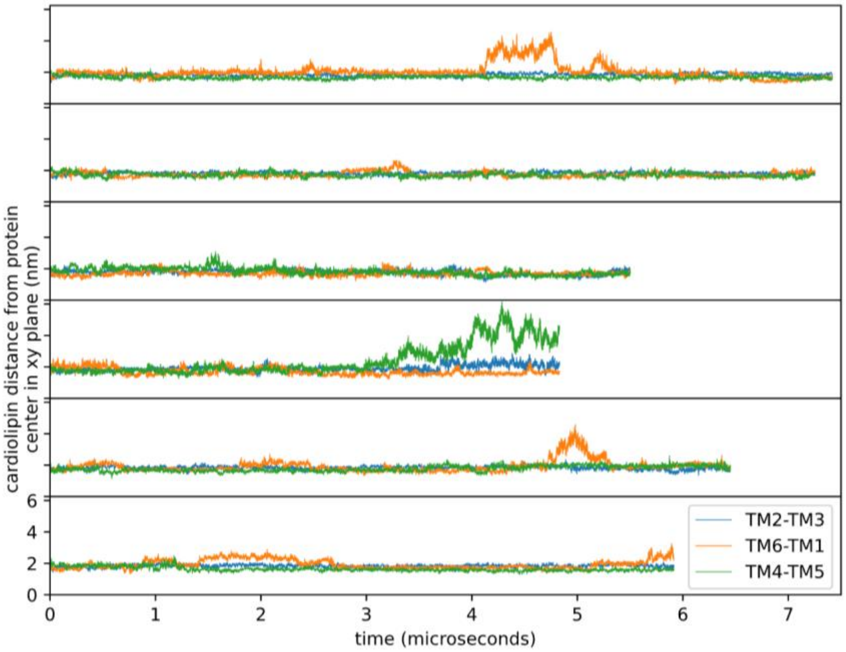

B. Cardiolipin distance from the protein as a function of water wire formation. Data points for the different runs are colored as in Fig. S3.

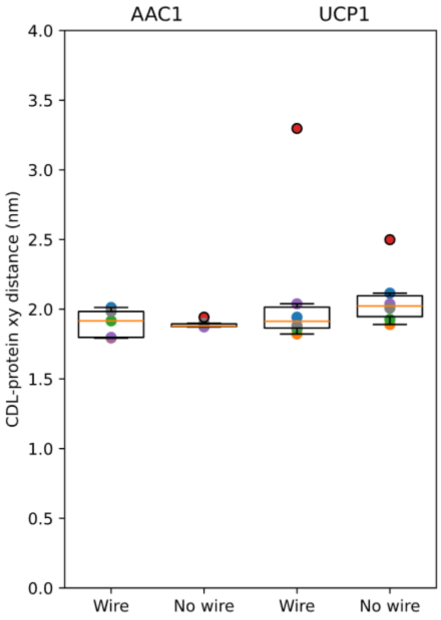

Figure S7. Distributions of maximum VMD PMEPot electrostatic potentials and the locations thereof. The y axis depicts probability density.

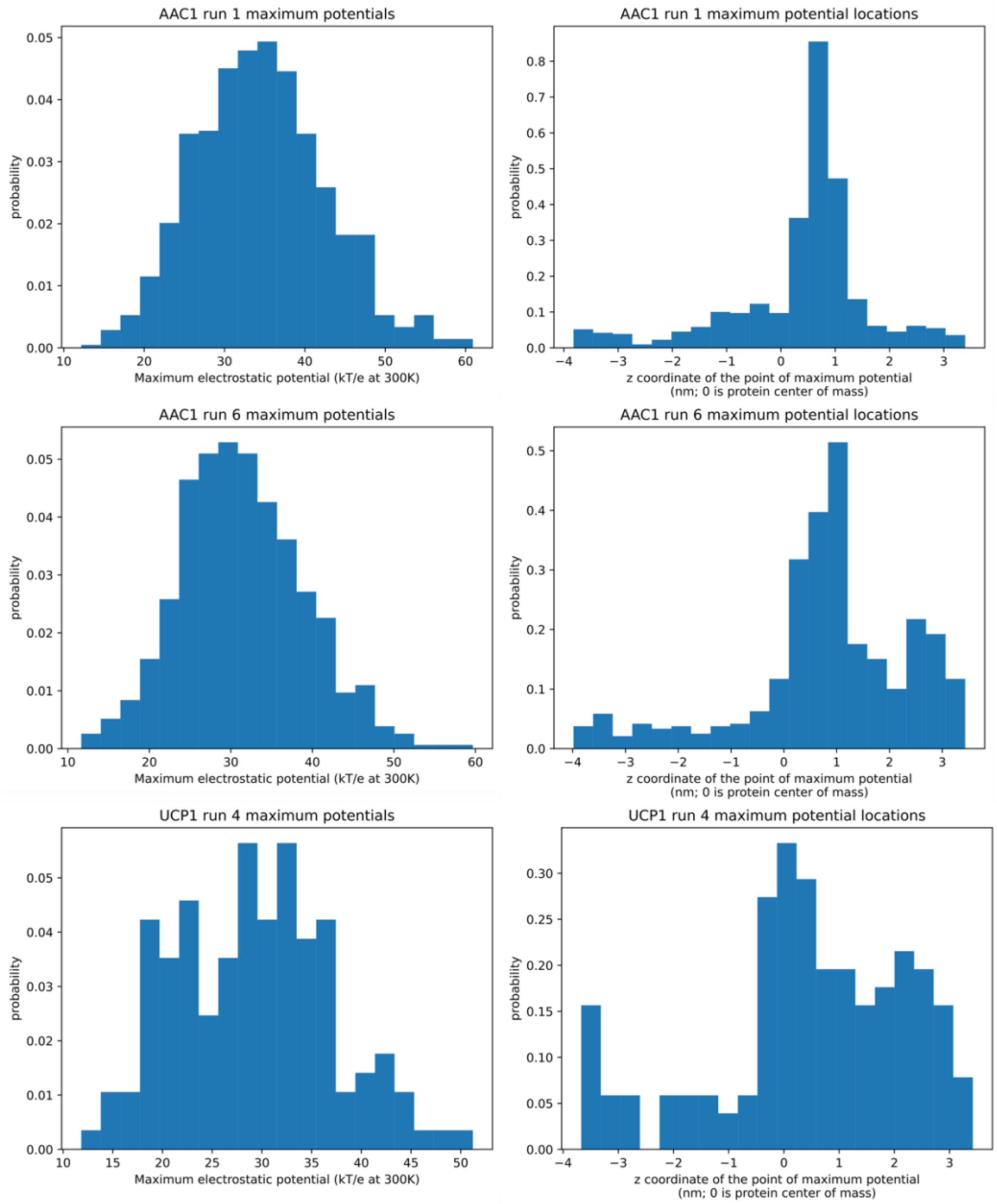

Figure S8. Example divergence of two instances of the same arachidonic acid molecule in parallel simulations started from the same configuration.

In AAC1 simulation runs 2 and 3, AA number 6, which starts far from the protein, binds between TM3 and TM4. In run 2, it reaches the TM3/TM4 site quickly and remains there for the rest of the simulation, while in run 3 it diffuses freely around the membrane for about three microseconds before eventually binding at the same site. The distance depicted in the plot below begins at 0 since both copies of AA 6 begin in the same configuration, rises as one copy binds at the TM3/TM4 site while the other remains in the membrane, and then falls as the other copy of AA 6 also binds at the TM3/TM4 site. The fact that the two AA had time to diverge to occupy distant parts of the box suggests that the eventual binding of both AA to the same site is a coincidence rather than an artifact of the starting membrane configuration.

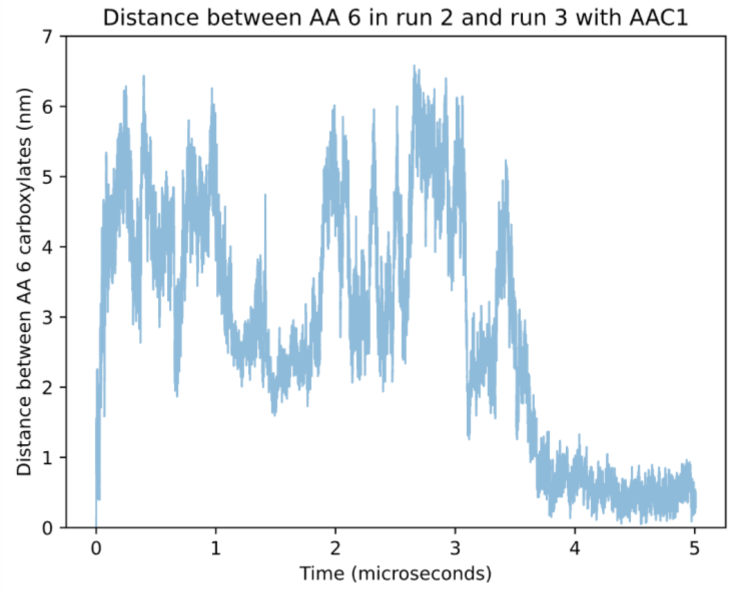

Supplement: Supplementary file 1 [file ijms-26-10504-s001.zip › ijms-3938355-supplementary.pdf]
